# Barriers to Healthy Eating and Influences on the Dietary Patterns and Eating Behaviours of 18–36‐Month‐Old Children in Ireland

**DOI:** 10.1111/jhn.70084

**Published:** 2025-06-25

**Authors:** Ben Leen Smith, Mairead E. Kiely, Elaine K. McCarthy

**Affiliations:** ^1^ Cork Centre for Vitamin D and Nutrition Research, School of Food and Nutritional Sciences University College Cork Cork Ireland; ^2^ INFANT Research Centre University College Cork Cork Ireland

**Keywords:** barriers to healthy eating, dietary patterns, eating behaviours, nutrition knowledge, picky eating, young children

## Abstract

**Background:**

Young children have high nutritional requirements relative to their size and energy intakes, yet inadequate nutrient intakes are widespread. Factors impacting the ability of caregivers to provide nutritionally adequate diets to young children are understudied.

**Objective:**

To evaluate key influences on the dietary patterns and eating behaviours of young children in Ireland.

**Methods:**

Parents and guardians with a child aged 18–36 months were invited to complete a self‐administered online survey. The 103‐question survey was delivered across 5 subsections (Socio‐Demographics, Parental Nutrition Knowledge, Parental Feeding Practice, Child Food Fussiness, Barriers to Healthy Eating and Dietary Patterns). Adherence (%) to current Dietary Guidelines for 1–5‐year‐olds and the Children's Food Pyramid were assessed using a food frequency approach to create an adherence score based on 7 components, including consumption of red and processed meat, fish, dairy, fruit and vegetables, confectionary and beverages.

**Results:**

We received 1158 responses, mostly from mothers (96.3%) born in Ireland (85.5%), of whom 80.1% had at least a primary degree. The mean (±SD) age of children was 26.2 ± 5.8 months and 54.6% were enrolled in an early years service, of which 74.3% provided food. The mean (±SD) dietary guideline adherence score among children was 55.5% ± 19.7%. The Children's Food Pyramid was recognised by 76.3% of parents and mean (±SD) nutrition knowledge score was 57.9% ± 14.6%, which was associated with dietary guideline adherence (*r* = 0.122, *p* < 0.001). Reported barriers to healthy eating were “*food fussiness”* (49%), “*time to prepare healthy foods”* (47%) and “*provision of unhealthy foods by caregivers outside the home”* (47%). Moderate to severe fussy eating was noted in 36% of children and food fussiness was associated with lower dietary guideline adherence (*r* = −0.172, *p* < 0.001). The children of respondents (13.1%) following restrictive diets (e.g., vegetarian, gluten‐free) had a lower than average dietary guideline adherence score (50.2% ± 18.3% *p* < 0.001).

**Conclusions:**

Many associated factors influence the dietary patterns of young children. Improved understanding of these influences may help to guide the design of targeted nutrition supports and education programmes for this age group.

## Summary

- Children demonstrated low adherence with national dietary guidelines, with particularly poor adherence to recommendations for processed meat, dairy and red meat.
- Despite a highly educated sample, parents lacked adequate nutrition knowledge, potentially impacting their children's diets. Increased nutrition knowledge was associated with better adherence to children's dietary guidelines.
- Food fussiness was the most frequently reported barrier to healthy eating, with over one‐third of children exhibiting moderate to severe fussy eating. Fussy eating was associated with decreased adherence to dietary guidelines.

## 1 Introduction

Young children have increased nutritional requirements relative to their energy requirements due to their rapid physical and neurological development [1, 2, 3]. Despite this, poor quality diets, characterised by high energy dense and low‐nutrient foods, are widespread among young children [4, 5]. Micronutrient deficiencies in early childhood can have detrimental and persistent effects on childhood development [2, 6, 7] and deficiencies are widespread among young children [4, 8, 9]. An estimated 56% of young children worldwide suffer from a deficiency in at least one of iron, zinc or vitamin A [8]. While often considered a problem primarily in low‐resource settings, 45% of young children in the United Kingdom (UK) were estimated to suffer from a deficiency in at least one of iron, zinc or vitamin A [8]. Inadequate intakes of iron and vitamin D were reported among 2‐year‐olds in Ireland; 20.8% had suboptimal iron status and 36% had low vitamin D status; where children not consuming voluntarily fortified foods had higher risks of inadequate intakes [9, 10, 11].

Young children may also be at risk of the double burden of malnutrition, due to increasing intakes of energy dense foods and rising rates of overweight and obesity [12, 13]. European data estimates that 29.5% of young children are overweight and 11.6% are obese [14]. Among 3‐year‐olds in Ireland, 18.3% were overweight and 5.2% were obese and there was a similar prevalence of overweight (23%) and obesity (10%) in the UK [15, 16]. Overweight and obesity in early childhood often tracks into adulthood and is associated with an increased risk of metabolic disease in later life [17, 18].

Establishing good nutrition practices and adequate dietary intakes in early childhood may create habits that persist into adulthood [19]. The drivers of dietary intakes in young children are complex, largely due to the dependence of this age group on their parents and external caregivers as the primary providers of food and meals. Young children's eating behaviours can be driven by a mirroring of parent's behaviours, as parental nutrition knowledge and feeding practices have also been shown to affect the foods provided [20, 21, 22, 23]. Individual behaviours such as food fussiness can also affect the quality of dietary intakes [24].

Currently, there are few parental nutrition knowledge assessments [25, 26], particularly exploring how parental nutrition knowledge may affect children's dietary patterns. This study aims to provide a comprehensive assessment of the dietary patterns, eating behaviours, parental feeding practices, parental nutrition knowledge and key barriers to healthy eating among 18–36‐month‐old children in Ireland.

## 2 Methods

Information on children's feeding behaviours, dietary pattens, perceived barriers to healthy eating, parental feeding practices and parental nutrition knowledge were collected using a self‐administered, web‐based survey (administered via *Qualtrics*) completed by parents/guardians of children aged 18–36 months.

### 2.1 Ethical Approval

This study was conducted in accordance with the guidelines laid down in the Declaration of Helsinki, with ethical approval granted by the UCC Social Research Ethics Committee (SREC) on April 3rd, 2023 (Log 2023‐053). All participants provided informed consent (electronically) before beginning the survey.

### 2.2 Survey Design

The survey consisted of 103 questions, delivered across 5 subsections: family demographics, parental nutrition knowledge, feeding practice and fussy eating, barriers to healthy eating and children's dietary patterns.

Section one included questions on the parent or guardian (referred to from here on as parent) and child age, sex and one question on adherence to restrictive diets (dietary pattern that excludes or limits specific foods or food groups), in addition to details on family size, child's birth order, marital status and education history. Parents were asked to indicate if their child was attending an early years service (EYS) and if so, whether this was on a part‐time or full‐time basis and to specify all types of service used. Parents could select one or more types of service. Full day care is defined as a structured service lasting > 5 h, with part‐time care lasting 3.5–5 h [27]. Parental nutrition knowledge was assessed across 14 questions, with 10 questions adapted from the general nutrition knowledge component of the Abridged Nutrition for Sport Knowledge Questionnaire [28]. These questions were selected for their relevance in assessing basic knowledge of the roles and sources of macronutrients and micronutrients. Questions specific to sport and exercise were removed. Results from this section were combined to calculate a total nutrition knowledge score.

Parental feeding practice was evaluated using the Child Feeding Questionnaire (CFQ) validated by Birch et al [29]. The CFQ uses a 5‐point Likert scale to quantify feeding practice across seven subsections: perceived responsibility for child's feeding (PR), perceived parental weight (PPW), perceived child weight (PCW), parental concern about their child becoming overweight (CN), control of food intake by restriction of foods (RST), pressure to eat more food despite hunger/satiety cues (PE) and monitoring of child's eating (MN). The minimum score in each section is 1 and the maximum score is 5, with higher mean (± SD) scores indicating a higher tendency for a given behaviour. Internal consistency across the CFQ sub scales was acceptable (Cronbach's alpha > 0.70), except for the PCW subscale which showed lower reliability (*α* = 0.56).

Additional feeding practices were assessed using a 5‐point Likert scale, including questions on the addition of sugar and salt to increase food acceptance, the presence of screens at mealtime, families eating meals at the same time, repeat food exposure and children's breakfast consumption. Food fussiness is a behaviour characterised by reduced dietary diversity, whereby children display a hesitancy to consume many familiar foods and reject many novel foods [24, 30]. The food fussiness scale from the validated Child Eating Behaviour Questionnaire (CEBQ) was used to assess child food fussiness [31]. This is a 6‐question scale with scores ≥ 3 indicating moderate to severe food fussiness [32]. The food fussiness scale had a Cronbach's alpha of 0.92 indicating strong reliability and internal consistency. Barriers to healthy eating were assessed using a 15‐statement questionnaire, with responses measured on a 5‐point Likert scale.

The dietary patterns of children were evaluated using food frequency style questions, that assessed adherence to the Food Safety Authority of Ireland (FSAI) 2020 Scientific Recommendations for Food Based Dietary Guidelines for 1–5‐year‐olds (outlined in Table 1 **)** and the Healthy Ireland Children's Food Pyramid [33, 34]. A dietary guideline adherence score was calculated based on seven subcomponents of the dietary guidelines, including consumption of red meat, processed meat, fish, dairy, fruit and vegetables, confectionary and beverages. Adherence was estimated by assessment of the frequency that servings from each food group were provided to children. The portion sizes provided by parents were not measured, however a standard portion as estimated by the healthy food pyramid was provided as guidance for parents in their completion of the questions. For example, “How often is a portion of vegetables served to your child? (i.e. one Serving = ½ cup of cooked vegetables, 3–4 carrot sticks)”. Children's supplement use was also assessed but not included as one of the seven components for the calculation of the dietary guideline adherence score.

**Table 1:** An overview of the dietary recommendations from the FSAI 2020 Scientific Recommendations for Food Based Dietary Guidelines for 1–5‐year‐olds [33] and the Healthy Ireland Food Pyramid [34].

Food group	Recommendation
Fruit and vegetables	Children in this age group (18–36 months old) should consume at least 2–5 servings of fruit, salad or vegetables per day.
Dairy	Children should consume at least 550 mL of milk or equivalent amounts of dairy products (three servings) per day.
Red meat	Children should consume a 30 g serving of fresh red meat, three times per week.
Fish	Children in this age group should consume a serving of fish (approximately 30 g) at least once per week and up to twice per week.
Processed meat	Unprocessed meats are preferable to processed meats which should be limited in this age group.
Beverages	Water and milk are the only drinks recommended for serving in this age group.
Confectionary	Foods such as confectionary, cakes, crips, biscuits and sugar‐coated breakfast cereals are not recommended for this age group.

### 2.3 Recruitment

Eligible participants were parents or guardians aged 18 years or older in Ireland, with a child aged between 18 and 36 months at the time of survey completion. Participants were primarily recruited through distribution of the survey link to pre‐school managers and through social media from April‐September 2023. All participants were presented with an online participant information sheet and provided informed consent electronically before survey completion. Participants were not required to complete every question and retained the right to withdraw from the study at any point before data submission, with the anonymity and confidentiality of participants ensured throughout the study. Participation was voluntary and no financial incentive or compensation was provided for completing this survey.

### 2.4 Statistical Analysis

Statistical analysis was carried out using SPSS for Microsoft Windows (IBM SPSS Statistics 28.0.1). Descriptive analysis and frequency tables were generated to analyse demographic and food frequency data. Nutrition knowledge, food fussiness and child feeding questionnaire scores were calculated individually for each respondent. Independent sample t‐tests were used to calculate differences in sample means across categorical variables. Chi‐square and fisher's exact tests were used to analyse the relationship between categorical variables. Pearson's correlation coefficients were used to examine the relationship between continuous variables. A *p* < 0.05 was considered significant. Cronbach's alpha was used to measure internal consistency across polytomous variables.

## 3 Results

### 3.1 Participant Characteristics

In total, 1158 parents with a mean (±SD) age of 36.1 ± 4.2 years completed the survey. Most parents were born in Ireland (85.2%) and had an average of 1.8 ± 0.9 children; the characteristics of study participants are summarised in Table 2. Children had a mean age of 26.2 ± 5.8 months, and most children were first born (59.8%). Over half (54.6%) were enrolled in EYS, of which 74.3% provided food. Of those enrolled in EYS, the most common service types were crèche (informal, care focused service) (65.2%), pre‐school (structured early education programme) (19.4%), childminder (11.7%) and full day care (11.0%).

**Table 2:** Participant characteristics (*n* = 1158).

Parent characteristics	% (*n*) or Mean ± SD
Sex–female	96.3 (1115)
Country of birth–Ireland	85.2 (987)
Employment status‐Employed (Full‐time)	69.2 (801)
Highest level of education ‐ Undergraduate degree or higher (≥ Level 7)	80.8 (936)
Relationship status–married	79.0 (915)
Adherence with restrictive diet	**13.1 (152)**
Vegetarian	32.3 (49)
Gluten‐free	23.7 (36)
Dairy‐free	19.7 (30)
Vegan	7.9 (12)
Child characteristics	
Age (Months)	26.2 ± 5.8
Sex–female	45.7 (529)
Adherence with restrictive diet	**9.5 (110)**
Dairy‐free	51.8 (57)
Vegetarian	11.8 (13)
Egg‐free	8.2 (9)
Reasons for restrictive diet	
Allergies	45.1 (46)
Intolerances	23.5 (24)
Parental beliefs	20.6 (21)

### 3.2 Children's Dietary Patterns

#### 3.2.1 Food Provision

**Figure 1:**
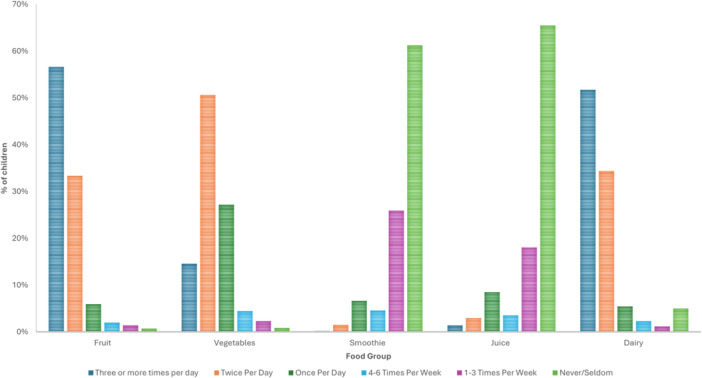
Frequency of fruit, vegetable, dairy and drink provision to children (*n* = 1009).

**Figure 2:**
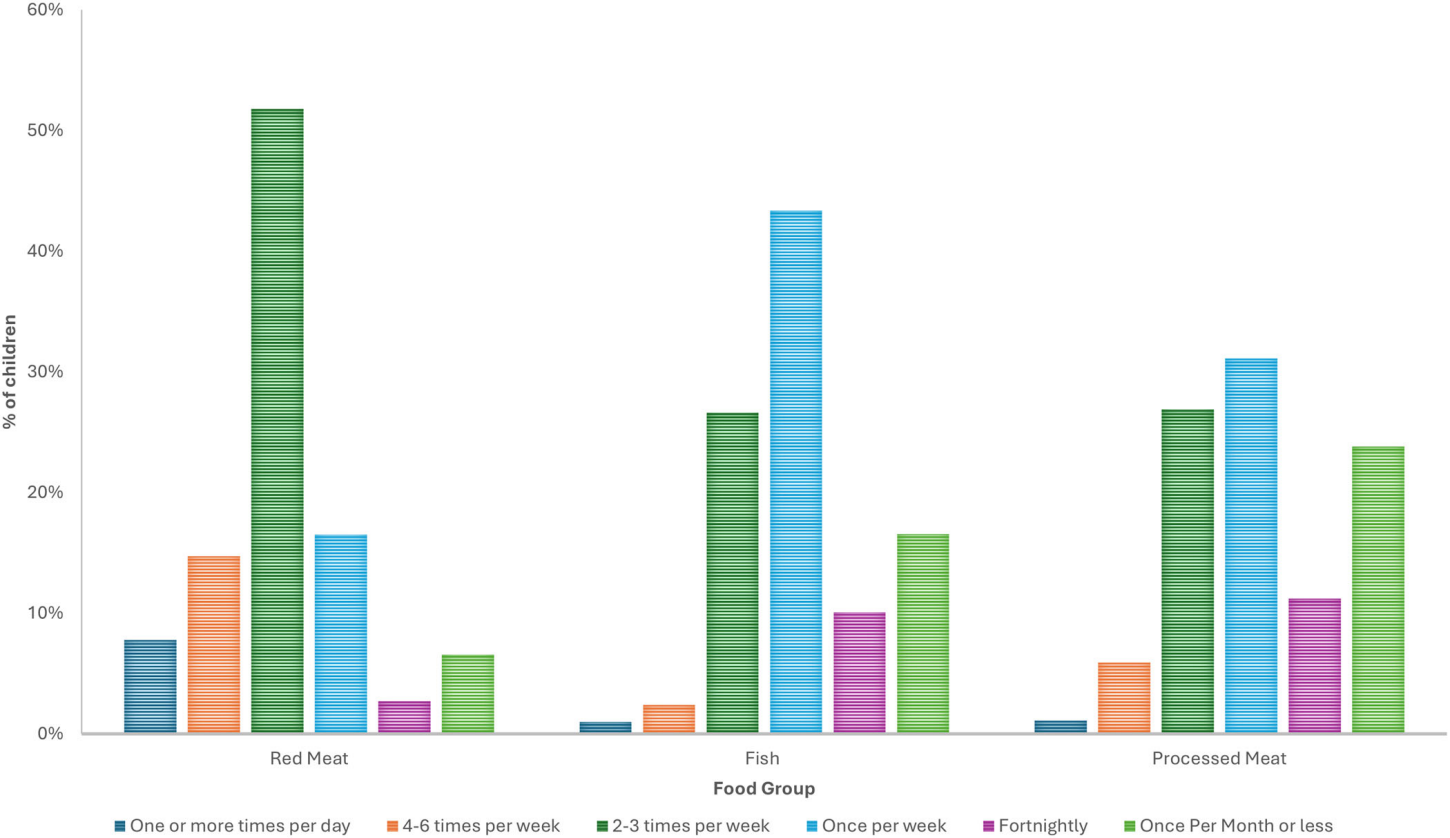
Frequency of red meat, fish and processed meat provision to children (*n* = 1009).

The frequency of fruit, vegetable and dairy provision to children is outlined in Figure 1, with the frequency of fish, red and processed meat provision in Figure 2. Less than 1% of parents served sugar sweetened beverages, while 29% served cordial (fruit drink concentrate). Confectionary was provided to children 3 or more times per week by 42.6% of parents. Nutritional supplements were used by 44.1% of children, including “multivitamins” (48.8%), “Vitamin D” (44.2%) and a “Probiotic” (12.6%).

#### 3.2.2 Adherence to Dietary Guidelines

**Figure 3:**
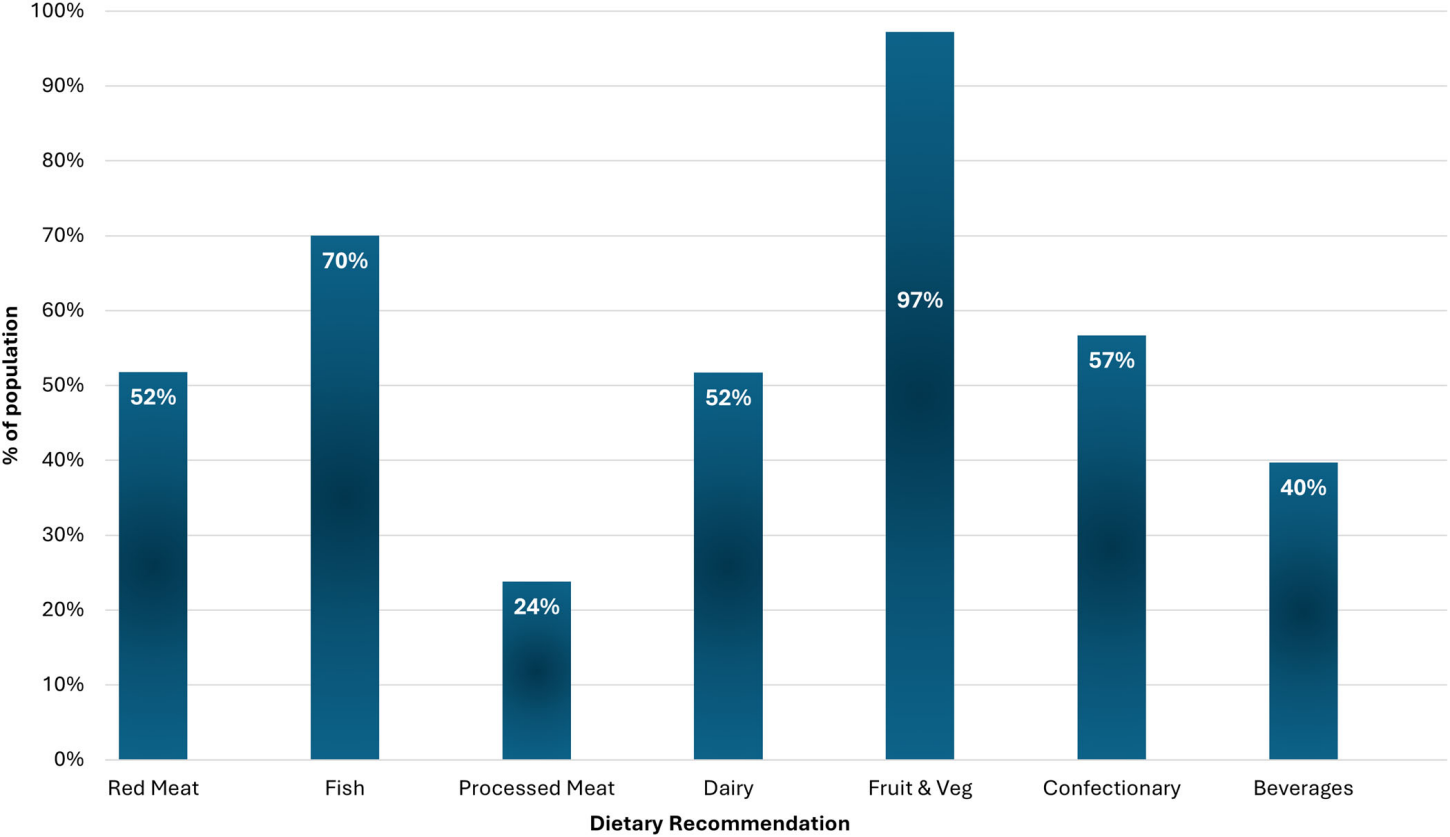
Adherence with individual components of the FSAI Dietary Guidelines for 1–5‐year olds (*n* = 1009).

Children had a mean (± SD) dietary guideline adherence score of 55.5% ± 19.7%. Adherence to the individual components of the dietary guidelines is presented in Figure 3.

A higher mean dietary guideline adherence score (56.3% ± 19.4% vs. 50.5% ± 19.2%, *p* = 0.001) was noted in children of parents that had completed a higher level of education (≥ Level 7 degree). Children attending EYS recorded lower adherence to dietary guidelines for processed meat (19.8% vs. 28.8%, *p* = 0.001).

First born children had a greater mean dietary guideline adherence score (57.1% ± 20.3% vs. 52.6% ± 18.0%, *p* < 0.001), with greater adherence to the dietary guidelines for processed meat (27.2% vs. 16.4%, *p* < 0.001) and confectionary (61.1% vs. 47.7%, *p* < 0.001) than non‐first born children. Compared to older children (≥ 24 months), younger children had greater adherence to the dietary guidelines for beverages (49.5% vs. 33.6%, *p* < 0.001), confectionary (70.7% vs. 49%, *p* < 0.001) and processed meat (33.2% vs. 17.8% *p* < 0.001).

Restrictive diets were followed by 13.1% of parents and 9.5% of children, most commonly dairy‐free, which accounted for over half (51.8%) of the restrictive diets followed by children. Parents primarily cited allergies (56.1%) or intolerances (43.9%) as reasons for dietary restriction. Vegetarian diets accounted for 32.3% of parent's and 11.8% of children's restrictive diets. Children of parents following restrictive diets recorded a lower mean dietary guideline adherence score (50.2% ± 18.3% vs. 56.4% ± 19.8%, *p* < 0.001), with lower adherence to dietary guidelines for red meat (38.4% vs. 53.6%, *p* < 0.001), fish (59.7% vs. 71.6%, *p* = 0.007) and dairy (30.9% vs. 55.3%, *p* < 0.001). In contrast, these children had greater adherence to the dietary recommendation for processed meat (37% vs. 21.8%, *p* < 0.001). Similarly, children adhering to a restrictive diet recorded a lower mean dietary guideline adherence score (48.3 ± 16.1% vs. 56.3 ± 19.9%, *p* < 0.002), with lower adherence to the guidelines for red meat (41.0% vs. 52.8%, *p* = 0.048), fish (57.5% vs. 71.2%, *p* = 0.016), dairy (18.3% vs. 55.0%, *p* < 0.001) and beverages (24.7% vs. 40.4%, *p* = 0.003) despite greater adherence to the guidelines for processed meat (34.8% vs. 22.6%, *p* = 0.01).

### 3.3 Parental Nutrition Knowledge and Feeding Practices

#### 3.3.1 Nutrition Knowledge

Parents had a mean (±SD) nutrition knowledge score of 57.9% ± 14.6% and the Healthy Ireland Children's Food Pyramid was recognised by 76.3%. Parents with a Level 7 degree (ordinary bachelor's degree) or higher recorded higher nutrition knowledge (59.0% ± 14.7% vs. 51.8% ± 13.0%, *p* < 0.001). Nutrition knowledge scores were positively correlated with children's dietary guideline adherence scores (*r* = 0.122, *p* < 0.001).

#### 3.3.2 Feeding Practices

**Figure 4:**
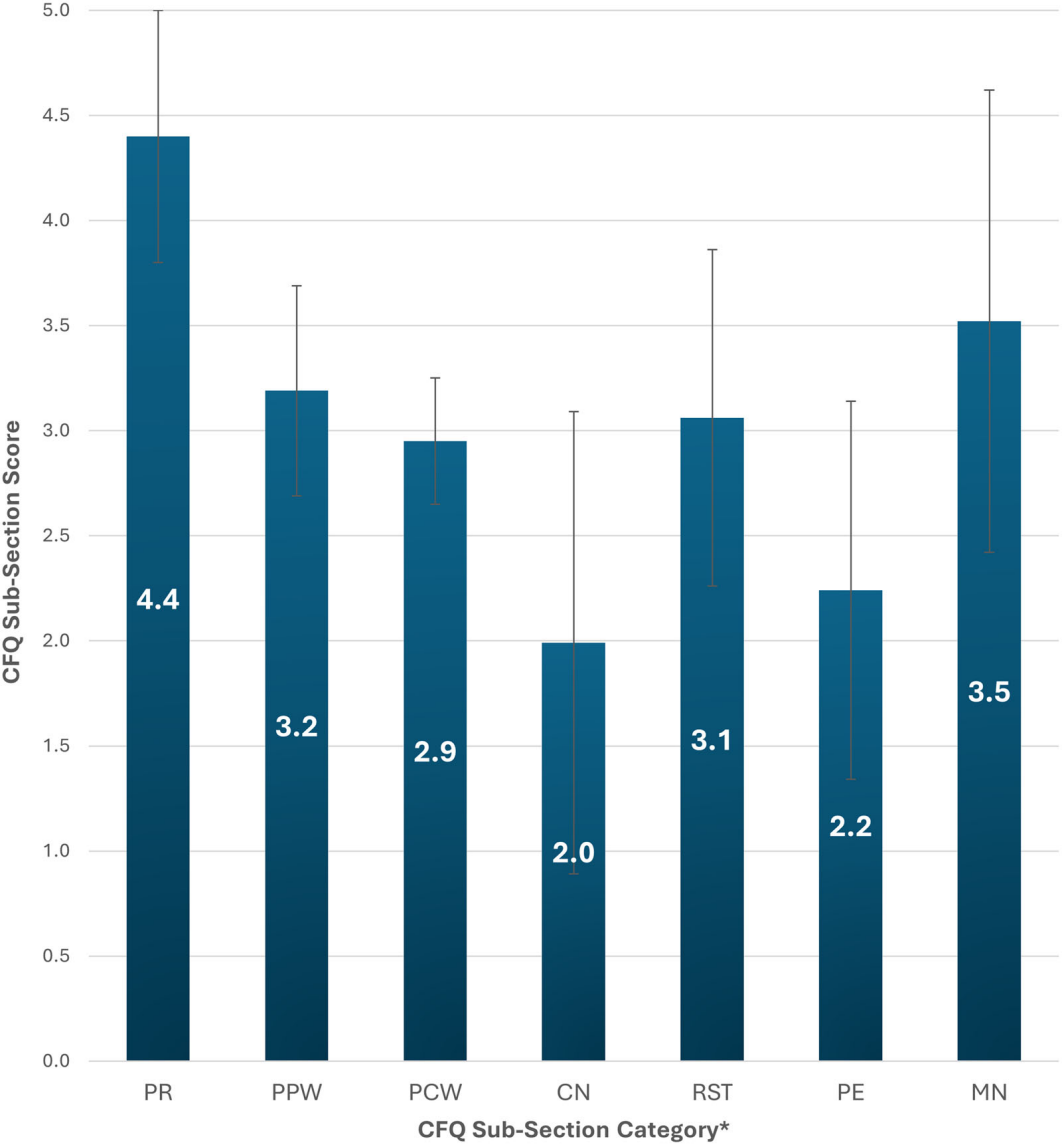
Mean (SD) scores for each subsection of the Child Feeding Questionnaire (n = 1073). Abbreviations for the CFQ subsections are given as follows: CN, parental concern; MN, monitoring; PCW, perceived child weight; PE, pressure to eat; PPW, perceived parental weight; PR, perceived responsibility; RST, restriction.

The scores for each subsection of the CFQ are outlined in Figure 4. Parents with a level 7 degree or higher recorded lower mean (±SD) scores in the perceived responsibility (4.4 ± 0.6 vs. 4.6 ± 0.6, *p* < 0.001), pressure to eat (2.2 ± 0.9 vs. 2.4 ± 1.0, *p* = 0.006) and monitoring (3.5 ± 1.1 vs. 3.7 ± 1.1, *p* = 0.05) subsections. Parents answering about their first born child indicated significantly higher concern about their child becoming overweight or obese (2.1 ± 1.1 vs. 1.9 ± 1.0, *p* = 0.006).

Lower dietary guideline adherence scores in children were correlated with higher parental restriction of children's dietary intake (*r* = −0.18, *p* < 0.001), exerting greater pressure to eat (*r* = −0.16, *p* < 0.001) and higher perceived parental weight (*r* = −0.10, *p* = 0.001). Higher dietary guideline adherence scores were correlated with greater parental monitoring (*r* = 0.09, *p* = 0.003). Higher nutrition knowledge was negatively correlated with perceived parental weight (*r* = −0.06, *p* = 0.043), parental concern (*r* = −0.06, *p* = 0.049), restriction (*r* = −0.065, *p* = 0.035), pressure to eat (*r* = −0.18, *p* < 0.001) and perceived responsibility (*r* = −0.11, *p* < 0.001) scores. Lower parental nutrition knowledge was noted in parents who used sweets as a reward (46.5% ± 13.7% vs. 58.0% ± 14.6%, *p* < 0.001), allowed access to screens (53.8% ± 13.7% vs. 59.0% ± 14.5%, *p* < 0.001) or whose children needed screens at mealtime (52.4% ± 12.6% vs. 58.6% ± 14.5% *p* = 0.002).

### 3.4 Barriers to Healthy Eating

**Figure 5:**
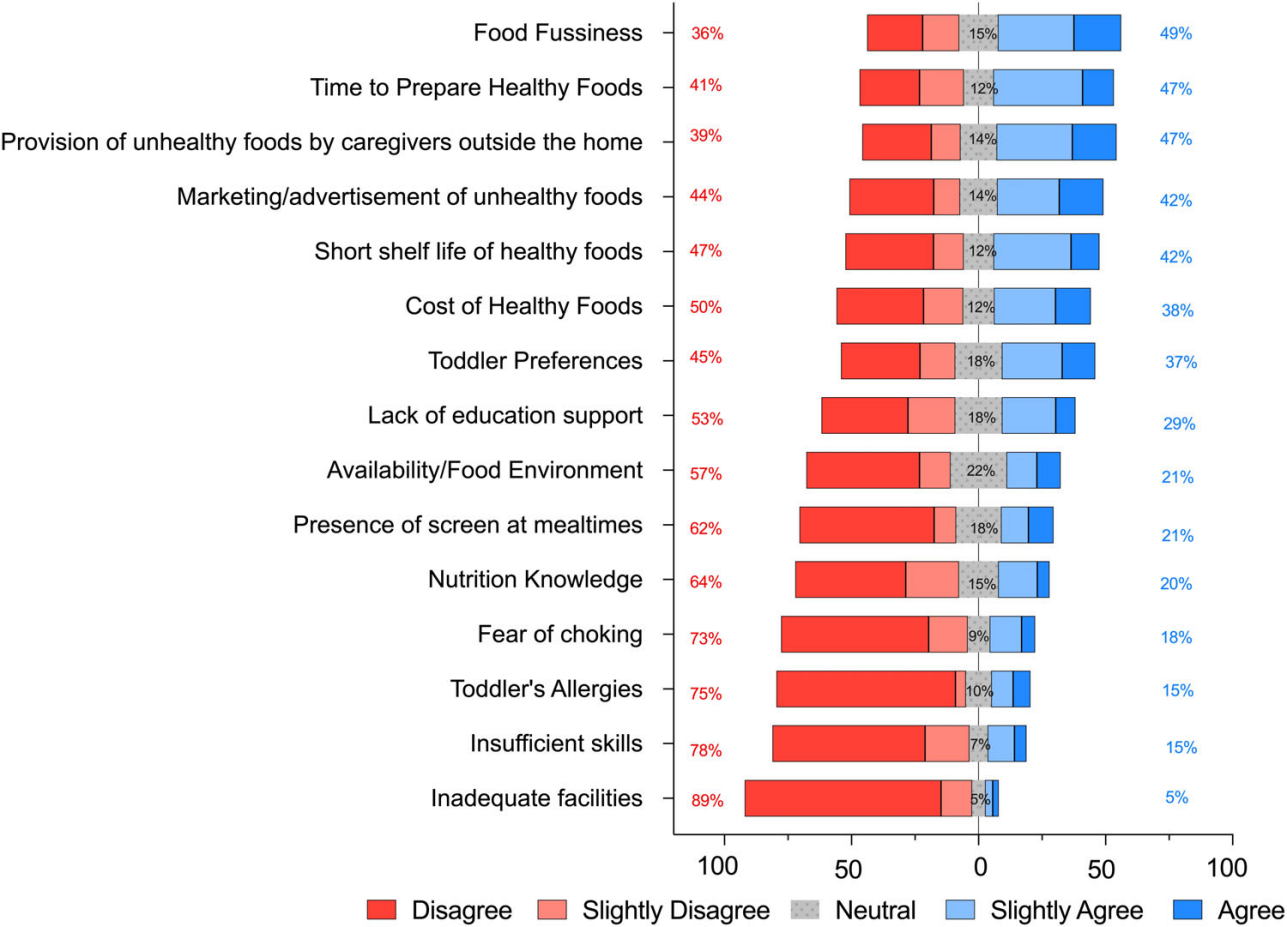
Perceived barriers to healthy eating in young children as identified by their parents (n = 1021).

Important barriers to healthy eating identified by parents were *food fussiness*, the *time to prepare healthy foods* and the *provision of unhealthy foods by external caregivers* (Figure 5). Children of parents who identified *cost* (53.9% ± 19.8% vs. 57.6% ± 19.3%, *p* = 0.005), *food preferences* (53.6% ± 19.1% vs. 59.1% ± 19.9%, *p* < 0.001), *food fussiness* (53.9% ± 19.6% vs. 58.6% ± 19.9%, *p* < 0.001) or *the short shelf life of healthy foods* (53.9% ± 20.0% vs. 57.4% ± 19.0%, *p* = 0.007) as barriers to healthy eating had lower mean (±SD) dietary guideline adherence scores.

Food fussiness was identified as a barrier to healthy eating by 49% of parents. Children had a mean (± SD) food fussiness score of 2.72 ± 0.83; 36% of children had scores indicative of moderate to severe food fussiness. Higher food fussiness was correlated with lower parental monitoring of dietary intakes (*r* = −0.08, *p* = 0.018), perceived child weight (*r* = −0.08, *p* = 0.012) and dietary guideline adherence score (*r* = −0.17, *p* < 0.001). A positive correlation was noted between food fussiness and parental restriction (*r* = 0.16, *p* < 0.001) and pressure to eat (*r* = 0.36, *p* < 0.001). Lower food fussiness scores were noted among children who met the dietary guidelines for red meat (2.6 ± 0.8 vs. 2.8 ± 0.9, *p* = 0.001) and confectionary (2.6 ± 0.8 vs. 2.8 ± 0.9, *p* < 0.001), and among children who consumed breakfast (2.7 ± 0.8 vs. 3.5 ± 1.0, *p* < 0.001), ate meals together with family (2.6 ± 0.8 vs. 3.1 ± 0.9, *p* < 0.001) and were repeatedly exposed to previously rejected foods (2.7 ± 0.8 vs. 2.9 ± 0.9, *p* < 0.001). Children who were allowed (3.2 ± 1.0 vs. 2.6 ± 0.8, *p* < 0.001) or needed screens at mealtimes (3.6 ± 1.1 vs. 2.7 ± 0.8, *p* < 0.001) had higher food fussiness scores.

When food preference was a reported barrier, children were less likely to adhere to the dietary guidelines for red meat (47.7% vs. 54.6%, *p* = 0.014), processed meat (21.6% vs. 28.0%, *p* = 0.042), fish (66.0% vs. 74.9%, *p* = 0.005), confectionary (51.1% vs. 61.8%, *p* = 0.002) and beverages (37.0% vs. 44.3%, *p* = 0.043). The time to prepare healthy foods barrier was associated with lower adherence to the guidelines for fish (67.6% vs. 74.6%, *p* = 0.026) and processed meat (21.1% vs. 27.5%, *p* = 0.033) and children of parents who identified cost as a barrier had lower adherence to the guidelines for fish (66.4% vs. 74.7%, *p* = 0.021) and dairy (46.4% vs. 56.9%, *p* = 0.002).

## 4 Discussion

This survey among a large sample of parents highlights several associated factors that influence the dietary patterns and behaviours of young children. Low adherence to dietary guidelines was underpinned by limited parental nutrition knowledge despite the overall well‐educated profile of this sample. Influences on dietary patterns included food fussiness, nutrition knowledge, cost, time, food preferences and parental feeding practices.

Overall compliance with the national dietary guidelines for 5‐year‐olds was low [33]. Inconsistencies in adherence to dietary guidelines were apparent, with high levels of adherence to the guidelines for fruit, vegetables and fish, but poor adherence with guidelines for processed meat, red meat, dairy and beverages. Poor compliance with recommendations for beverages was highlighted by high rates of smoothie and fruit juice consumption. Fruit juice intake has previously been noted as the greatest contributor to non‐milk sugar intakes in Irish children [13], which has been implicated in the development of overweight, obesity and dental caries within this age group [35].

The most prominent barrier to healthy eating reported by parents was food fussiness, defined as a behaviour characterised by reduced dietary diversity and hesitancy to consume novel foods [24]. Over a third of children had food fussiness scores indicative of moderate to severe food fussiness, compared to 19.5% of children in a similar assessment in France [30]. Higher food fussiness scores were associated with lower adherence to dietary guidelines, which is consistent with previous investigations [24]. Lower food fussiness was noted in children who ate meals together with family and were repeatedly offered previously rejected foods, which is consistent with studies in the UK [24, 36, 37]. Higher pressure to eat and the presence of screens at mealtimes were associated with higher food fussiness here and elsewhere [38], while repeat exposure to previously rejected foods was associated with decreased food fussiness. The relationship between these feeding practices and behaviours with food fussiness provides guidance for the design of targeted food fussiness supports [39]. Whilst not assessed in our study, parental food fussiness has previously been associated with child food fussiness [40] which may suggest that unresolved childhood food fussiness continues to persist in future generations. Given the impact of food fussiness on dietary patterns within our sample, future research in this area should focus on combined assessments of both parents and children.

Low parental nutrition knowledge in our sample highlights potential issues in the communication and understanding of dietary guidelines [33], which is consistent with other reports among adults [25]. In our study, better nutrition knowledge among parents was associated with higher adherence to the dietary guidelines in their children. This highlights a need for more impactful nutrition education supports and tools to improve the communication and translation of dietary guidelines to families. Tools such as the food pyramid alone are insufficient to improve children's dietary intakes. Children's food choice is largely influenced by parental attitudes, skills and feeding practices [41, 42, 43]. Lower nutrition knowledge was associated with practices such as higher use of food as a reward, use of screens at mealtime and restriction of dietary intakes. Gaps in parental nutrition knowledge could be addressed similarly to previous education interventions delivered in‐person and online [44, 45]. De Bock et al designed a structured, in‐person education program delivered through pre‐schools resulting in improved fruit and vegetable intake among children [44]. Alternatively, the Time2bhealthy study delivered an online education programme, with fortnightly follow up emails, leading to improvements in parental feeding practices and children's discretionary food intake [45]. These interventions highlight the potential to improve nutrition knowledge and feeding practice together, which may positively influence children's dietary patterns.

The cost of healthy foods is increasingly becoming a barrier to the provision of a healthy diet to children [46]. In 2010, 23%–26% of parents with 24–36‐month‐old children identified cost as a barrier to healthy eating in contrast to 38% of our sample [13]. Considering the 12.7% increase in the consumer price index (CPI) rate for food and non‐alcoholic beverages in Ireland since July 2021, with a 20.3% rise in the UK in the same period [47, 48], it is likely that this issue will become increasingly important. Coupled with the prominence of “the short shelf life of healthy foods” as a barrier to healthy eating, this may lead to a reliance on more convenient or processed foods. The health disparity between higher and lower income families is multi‐faceted and may be cyclical in nature [49, 50]. Children with parents who had completed a higher level of education appeared to have slightly better nutrition knowledge and were more likely to adhere to the dietary guidelines. Education level has previously been indicated as the most consistent indicator of socioeconomic status [51]. Families of lower socioeconomic status have previously been shown to have increased food insecurity, less access to health resources and suffer from greater rates of premature mortality and morbidity [52, 53, 54]. Individuals from lower socioeconomic positions also appear to be at greater risk of exposure to patterns and behaviours that may mediate disease risk (i.e., obesity, smoking) [52, 55, 56]. Poor nutrition knowledge may exacerbate this health disparity, while highlighting areas for focused intervention. The provision of targeted nutrition supports and subsidies to parents of lower socioeconomic status, including food voucher schemes and education programmes [57, 58, 59], may help reduce the burden of this health disparity.

Foods provided by external caregivers were identified as another major barrier to healthy eating by parents. Over half of our participants attended some type of EYS, with most of these services providing food to the children in attendance. Lower adherence to the dietary guideline for processed meat was noted in the children attending EYS, however as the foods provided by EYS were not assessed in our study, we cannot determine if poor adherence was due to these services. Previous data has indicated that processed meats were noted on the menu of 79.6% of preschools in Ireland [60]. TUSLA, the Irish governing body of EYS has set out a regulatory framework for services to adhere to, with policies governing feeding practice, healthy eating and the foods provided to children [61, 62]. Whilst these policies state that “processed foods should be used sparingly”, our results may indicate a need for increased governance and monitoring of healthy eating policy implementation [61, 62, 63]. It is plausible that lower adherence to the dietary guideline for processed meat consumption in children attending EYS may also reflect the time constraints experienced by parents in full‐time employment. The time to prepare healthy foods was the second most prominent barrier to healthy eating in our study and children whose parents identified time as a barrier were less likely to meet the dietary guideline for processed meat. This aligns with previous Irish data where 39% of parents agreed that convenience was a constraint to the delivery of a healthy diet [13].

While data is provided on adherence to the national dietary guidelines, it only provides an estimate of adherence based on the frequency that food groups were provided to children. It does not account for portion size estimation or appropriately quantify intakes. The use of questions adapted from the general nutrition knowledge section of the ANSK‐Q, initially designed for an athlete population is a limitation of this assessment. Despite removing sport specific questions, insufficient measurement tools highlight the need to develop a reliable, validated tool to assess parental nutrition knowledge. Compared to the Central Statistics Office estimate of ~60% of adults under the age of 45 years having a tertiary qualification, a higher proportion of parents in our sample had a third level education, which limits the generalizability of these results [64]. Our study provides a novel assessment of parental feeding practices, parental nutrition knowledge, children's eating behaviour and children's dietary patterns and provides one of the first large scale assessments of both parental nutrition knowledge and feeding practices within Ireland.

## 5 Conclusion

The factors that influence children's dietary intakes and eating patterns are closely related and may influence each other. This study among well‐educated respondents reports low adherence to dietary guidelines associated with poor parental nutrition knowledge and unhealthy feeding practices. High levels of food fussiness were noted among children, which was also associated with low adherence to dietary guidelines. Improved parental education and skills development may help to combat high levels of food fussiness and promote healthy eating among young children.

## Author Contributions

Ben Leen Smith designed the survey with guidance from Elaine K. McCarthy and Mairead E. Kiely. Ben Leen Smith analysed the data. Ben Leen Smith drafted the manuscript, with inputs and revisions from Elaine K. McCarthy and Mairead E. Kiely. All authors reviewed and approved the final version of the manuscript.

## Ethics Statement

This study was approved by the UCC Social Research Ethics Committee (SREC) on April 3rd, 2023 (Approval Number: Log 2023‐053).

## Conflicts of Interest

The authors have no conflicts of interest to declare.

## 1 Peer Review

The peer review history for this article is available at https://www.webofscience.com/api/gateway/wos/peer-review/10.1111/jhn.70084.

## 2 Transparency Declaration

The lead author affirms that this manuscript is an honest, accurate and transparent account of the study being reported. The reporting of this study is compliant with STROBE guidelines. The lead author affirms that no important aspects of the study have been omitted and that any discrepancies from the study as planned have been explained.

## Data Availability

The data that support the findings of this study are available on request from the corresponding author. The data are not publicly available due to privacy or ethical restrictions.
